# Supplementary material for: POMK regulates dystroglycan function via LARGE1-mediated elongation of matriglycan
Source: eLife. 2020 Sep 25;9:e61388. doi: 10.7554/eLife.61388 (PMC7556876; doi:10.7554/eLife.61388)
Supplement: Transparent reporting form [file elife-61388-transrepform.docx]

***eLife’s* transparent reporting form**

We encourage authors to provide detailed information *within their submission* to facilitate the interpretation and replication of experiments. Authors can upload supporting documentation to indicate the use of appropriate reporting guidelines for health-related research (see [EQUATOR Network](http://www.equator-network.org/%20)), life science research (see the [BioSharing Information Resource](https://biosharing.org/)), or the [ARRIVE guidelines](http://www.plosbiology.org/article/info:doi/10.1371/journal.pbio.1000412) for reporting work involving animal research. Where applicable, authors should refer to any relevant reporting standards documents in this form.

If you have any questions, please consult our Journal Policies and/or contact us: [editorial@elifesciences.org](mailto:editorial@elifesciences.org).

**Sample-size estimation**

- You should state whether an appropriate sample size was computed when the study was being designed
- You should state the statistical method of sample size computation and any required assumptions
- If no explicit power analysis was used, you should describe how you decided what sample (replicate) size (number) to use

Please outline where this information can be found within the submission (e.g., sections or figure legends), or explain why this information doesn’t apply to your submission:

- Appropriate sample sizes were decided based on previously published studies, and these have been referenced in the Methods.
- The number of mice to be used and the number of replicates to be performed were based on previously published studies, and these are described in the Methods under “Collection of Embryos and Microinjection”.

**Replicates**

- You should report how often each experiment was performed
- You should include a definition of biological versus technical replication
- The data obtained should be provided and sufficient information should be provided to indicate the number of independent biological and/or technical replicates
- If you encountered any outliers, you should describe how these were handled
- Criteria for exclusion/inclusion of data should be clearly stated
- High-throughput sequence data should be uploaded before submission, with a private link for reviewers provided (these are available from both GEO and ArrayExpress)

Please outline where this information can be found within the submission (e.g., sections or figure legends), or explain why this information doesn’t apply to your submission:

- Biological replicates: For mice, each biological replicate represents a different pair of mice (i.e. one biological replicate is one pair of mice: 1 control mouse and 1 knockout mouse. Subsequent biological replicates represent different pairs of animals).
- For cell experiments, each biological replicate represents separate plates of cells (1 plate of control, 1 plate of knockout, and 1 plate of double knockout cells constitute one biological replicate).
- For all experiments, 3 biological replicates were performed, unless otherwise indicated.
- Technical replicates (using the same sample multiple times) were not performed, unless indicated.
- The number of replicates required was based on previously published studies using the described techniques.
- The number of independent biological replicates performed can be found in the figure legend. Additional information on numbers of mice used can be found in Methods under “Collection of Embryos and Microinjection” or “Statistics”.
- No outliers were encountered.
- No data were excluded.
- No high-throughput sequence data were included in the manuscript.

**Statistical reporting**

- Statistical analysis methods should be described and justified
- Raw data should be presented in figures whenever informative to do so (typically when N per group is less than 10)
- For each experiment, you should identify the statistical tests used, exact values of N, definitions of center, methods of multiple test correction, and dispersion and precision measures (e.g., mean, median, SD, SEM, confidence intervals; and, for the major substantive results, a measure of effect size (e.g., Pearson's r, Cohen's d)
- Report exact p-values wherever possible alongside the summary statistics and 95% confidence intervals. These should be reported for all key questions and not only when the p-value is less than 0.05.

Please outline where this information can be found within the submission (e.g., sections or figure legends), or explain why this information doesn’t apply to your submission:

- Statistical analysis methods, samples sizes, and exact p-values for each experiment can be found in the figure legend.
- Any p-values include the exact value, whenever possible.
- Raw data are displayed whenever possible.
- More detailed methods, such as how statistical testing was performed and what error bars indicate, can be found in the Methods section under the relevant experimental technique or in the section of the Methods titled “Statistics”.
- Human skeletal muscle studies were done on a small biopsy that had very limited material.
- For human skeletal muscle POMK activity, only one replicate was performed due to the limited amount of sample available for the assay.
- For human skeletal muscle B4GAT1 activity, two technical replicates were performed (B4GAT1 activity from the same muscle lysate sample was measured twice).
- No statistical testing could be done for human skeletal muscle POMK and B4GAT1 activity due to the number of replicates performed.

(For large datasets, or papers with a very large number of statistical tests, you may upload a single table file with tests, Ns, etc., with reference to sections in the manuscript.)

**Group allocation**

- Indicate how samples were allocated into experimental groups (in the case of clinical studies, please specify allocation to treatment method); if randomization was used, please also state if restricted randomization was applied
- Indicate if masking was used during group allocation, data collection and/or data analysis

Please outline where this information can be found within the submission (e.g., sections or figure legends), or explain why this information doesn’t apply to your submission:

- Samples were either control or knockout and were based on the genotype of the mouse or cell lines, which were known beforehand.
- We have not performed any studies requiring therapeutic intervention.
- Randomization was not used, as this was not relevant to our study design.
- Masking was not used, as this too was not relevant to our study design.

**Additional data files (“source data”)**

- We encourage you to upload relevant additional data files, such as numerical data that are represented as a graph in a figure, or as a summary table
- Where provided, these should be in the most useful format, and they can be uploaded as “Source data” files linked to a main figure or table
- Include model definition files including the full list of parameters used
- Include code used for data analysis (e.g., R, MatLab)
- Avoid stating that data files are “available upon request”

Please indicate the figures or tables for which source data files have been provided:

- Figure 6-figure supplement 2
